# How does multimorbidity affect middle-aged adults? A cross-sectional survey in the Singapore primary healthcare setting

**DOI:** 10.1186/s12875-020-01262-2

**Published:** 2020-09-14

**Authors:** Sai Zhen Sim, Hui Li Koh, Sabrina Poay Sian Lee, Doris Yee Ling Young, Eng Sing Lee

**Affiliations:** 1 Clinical Research Unit, National Healthcare Group Polyclinics, 3 Fusionopolis Link, Nexus@one-north. South Tower, # 05-10, Singapore, 138543 Singapore; 2 Division of Family Medicine, Yong Loo Lin School of Medicine, National University of Singapore, NUHS Tower Block, 1E Kent Ridge Road Level 11, Singapore, 119228 Singapore

**Keywords:** Multimorbidity, Health-related quality of life, Sociodemographic, Middle-aged, Primary care

## Abstract

**Background:**

Multimorbidity is of increasing prevalence and importance. It has been associated with poorer health-related quality of life (HrQoL) especially in the elderly population. Despite substantial multimorbidity in the middle-aged population, defined as those aged between 40-64 years old, there is a paucity of research investigating the impact of multimorbidity in this population. This study aimed to investigate the association between multimorbidity and HrQoL in the middle-aged primary care population in Singapore.

**Methods:**

A cross-sectional study was conducted at a primary care centre in Singapore. Interviewer-administered questionnaires were used to collect data regarding the participants’ sociodemographic characteristics, chronic conditions, and HrQoL, as measured by the EuroQol five dimensions 3-levels questionnaire (EQ5D). We defined multimorbidity as the presence of three or more conditions, out of a list of 14 chronic conditions. The associations between multimorbidity and the components of the EQ5D were assessed using multivariable regression analyses.

**Results:**

The study included 297 participants, aged 40–64 years, of which 124 (41.7%) had multimorbidity. After adjusting for sociodemographic factors, participants with multimorbidity had significantly lower EQ5D UI, (β-coefficient − 0.064 (C.I -0.125, − 0.003), *p* = 0.04), but not significantly lower EQ5D VAS, (β-coefficient − 0.045 (C.I 0.102, 0.012), *p* = 0.12). Additionally, participants with multimorbidity had higher odds (OR = 2.41, *p* = 0.01) of reporting problems due to pain/discomfort.

**Conclusion:**

Multimorbidity was not significantly associated with the overall health state, as measured by the EQ5D VAS, in middle-aged primary care patients. However, it was associated with the EQ5D UI which is a composite measure of five specific domains of HrQoL. Specifically, there was a statistically significant association between multimorbidity and the pain domain. Further studies are required to understand the relationship between multimorbidity and pain to enable physicians to better manage pain and HrQoL in this population.

## Background

Multimorbidity, defined as the presence of multiple chronic conditions without a specific index disease, [1] is prevalent in primary care [2, 3]. About 30 to 58% of middle-aged patients have multimorbidity, [2–4] and the absolute number of these patients may even exceed that of elderly patients [3]. A survey of the general population in Singapore found that 35% of middle-aged participants had multimorbidity [5] and although there are no official estimates in the Singapore primary care setting, the figure is likely to be higher. The prevalence of multimorbidity rises steeply in midlife and plateaus in those aged 75 years and above, [2] and this may be contributed by the growing emphasis on screening and early detection of chronic diseases. In fact, many countries have national health screening programmes [6–8] targeting the middle-age (40–64 years old) population. Thus there is an urgent need for healthcare professionals and policy makers to understand how multimorbidity affects this population.

People with multimorbidity have poorer HrQoL, [9–11] higher healthcare costs and utilization, [12, 13] higher mortality, [14] and reduced work productivity and working performance [15]. However, the impact of multimorbidity may be different across various age groups. While many studies have focused on the elderly, [16–18] few have focused on middle-aged adults. Younger people may have poorer HrQoL compared to the elderly, possibly due to the lower health expectations of older people or their ability to adapt better to lifestyle changes imposed by adverse health events [9, 11]. Midlife is the time when chronic illnesses start to surface and often take adults by surprise [19]. Work also plays a large role in the lives of middle-aged adults and is central to their identity [20]. While the midlife period may reflect the peak in professional attainment and earning for many, it is also a period when adults face multiple stresses and burdens such as medical bills, financial loans, caring for dependents, and bereavement [19]. There is an association between stress and self-rated health in midlife, those with poorer health ratings report more stress and less satisfaction in life [21]. By understanding how the co-existence of multiple chronic conditions affects middle-aged patients, suitable interventions can be designed to improve patient care and satisfaction. We aimed to describe the association between multimorbidity and HrQoL in the middle-aged primary care population in Singapore.

## Methods

A cross-sectional study was conducted in August 2017 at Hougang Polyclinic, which is part of a bigger network of clinics under the National Healthcare Group Polyclinics (NHGP). Hougang polyclinic is a large public primary care centre with approximately 140 healthcare providers including physicians, nurses, pharmacists, and allied health professionals. It provides a comprehensive range of healthcare services, including health screening, treatment for acute and chronic medical conditions, women and child health services, as well as dental care. The polyclinic serves the Hougang Township, the fifth largest township in Singapore, with approximately 223,010 residents of which 40% are middle-aged [22].

There is no strict definition of “middle-age” and studies have defined it as age between 40-65 years [23] or 45–64 years [3, 5, 24] In our study, the lower age limit was defined as 40 years old as that is also the minimum age at which general health screening is recommended [6]. According to the Singapore census those aged 65 years and above are considered elderly [22] and hence the upper limit of our middle-age range was defined as 64 years old.

With assistance from the NHGP Office of Clinical Informatics and clinic operations staff, we obtained the daily lists of middle-aged patients (40–64 years old) with physician appointments. The appointments were either scheduled in advance for regular review of their chronic conditions or scheduled on the same day as a walk-in appointment. Potential participants were selected in a systematic randomized manner based on the time and type (regular review or as walk-in) of appointment. They were then approached and screened for eligibility. The inclusion criteria were a) aged 40–64 years, b) had at least one or more chronic condition(s) out of a predetermined list of 14 chronic conditions, c) consented to access of electronic medical records for data collection, and d) spoke and understood any one of the three main languages in Singapore: English, Mandarin or Malay. Participants were excluded if they were non-communicative, unable to give consent, or if they did not complete the survey. Our study focused on how having chronic conditions affected one’s HrQoL and we also excluded those with no chronic conditions as they often present at our polyclinic with acute self-limiting conditions [25] which can transiently affect their HrQoL.

Recruitment and consent-taking were done by trained interviewers, including a research assistant, two medical students, and the principal investigator. The interviewer-administered questionnaires were conducted in a quiet area in the polyclinic.

### Definition of multimorbidity

We defined multimorbidity as the presence of three or more chronic conditions. Although many studies [3, 5, 9, 10] and organisations [26, 27] have used a cut-off of two or more chronic conditions to define multimorbidity, some studies [11, 18] have used a higher cut-off of three or more conditions. Holzer et al. [28] found a close relationship between the estimated prevalence of two or more conditions and that of three or more conditions, and that both definitions of multimorbidity also gave the same information on prevalence. In an unpublished cross-sectional study [29] of 787, 466 primary care patients in Singapore, the prevalence of multimorbidity in the middle-aged patients was 45.3% when the cut-off was two or more conditions. When the cut-off was three or more conditions, the prevalence decreased to 28.5% [29] For our study, using a higher cut-off to define multimorbidity can better identify patients with increased needs [2] and this is more meaningful in our setting*.*

Although there is no standardised definition of multimorbidity, using a list of at least 12 chronic conditions resulted in little variation in prevalence estimates of multimorbidity [2] Thus, we used a list of 14 chronic conditions to define multimorbidity: diabetes mellitus, hypertension, lipid disorder, neurological conditions, respiratory diseases, psychiatric conditions, cancer, chronic kidney disease, heart diseases, arthritis, back/neck problems, gastrointestinal diseases, thyroid disease and physical disability. This list was previously used by Quah et al. [16] to measure multimorbidity in elderly patients at a primary healthcare setting and was derived from the Singapore Mental Health Study [30] Participants were asked to report if they had any of the chronic conditions listed above, as told to them by a registered physician. In this study, the number of chronic condition(s) was categorized dichotomously, distinguishing those with one or two conditions from those with three or more conditions i.e. with multimorbidity.

### Measurement of health-related quality of life

HrQoL was measured by EQ5D-3 L questionnaire, [31] which has been validated locally, [32–34] and is available in the three most spoken languages in Singapore- English, Mandarin and Malay. The EQ5D consists of two components. The first component is the health-state Utility Index (UI). It measures five dimensions of HrQoL (mobility, self-care, usual activities, pain/discomfort and anxiety/depression) on a three-point severity scale (no problems, moderate problems or extreme problems). The Singapore time trade-off values were used to convert the information into UI scores, with − 0.790 being the worst health state and 1.000 being the best health state. The second component of the EQ5D is the visual analogue scale (VAS) which consists of a scale from 0 to 100. It is used to assess self-perceived global levels of health, with 0 representing the worst imaginable health state and 100 the best imaginable health state. Participants were asked to select a number on the VAS, which best represented their global health state for that day.

### Sociodemographic variables

The sociodemographic variables collected included age, sex, ethnicity, main spoken language, marital status, education level, work status, monthly household income (in Singapore dollars), type of dwelling, home ownership, and living arrangement. With regards to the type of dwelling, the options are more varied in view of Singapore’s unique housing landscape where the majority of the population live in subsidised housing provided by the Housing Development Board (HDB). The size and value of these apartments correspond to the number of rooms stated. In addition, there are hybrids of public and private housing such as the Executive Condominiums and Housing Urban Development Company apartments that cost more than the usual HDB apartments. The minority of the population stay in private housing that includes private condominiums and landed properties [22].

### Study sample

Sample size was calculated by assuming a Pearson’s coefficient of − 0.2, which was derived from the Spearman coefficient of the association between EQ5D UI and the count of chronic conditions reported in H Radner et al. [35] With alpha of 0.05 and power (1-beta) of 80%, the estimated sample size was 194. Assuming 30% refusal and incomplete data, the final calculated sample size was 278.

### Statistical analysis

The sociodemographic characteristics, number of chronic conditions, and EQ5D states of the study population were analysed descriptively. Means with standard deviations were calculated for continuous variables, while frequencies and percentages were computed for categorical variables.

A generalised linear model with log link function was used to analyse the associations between multimorbidity and each of the two components (UI and VAS) of the EQ5D, producing regression coefficients with 95% confidence intervals. Binary logistic regression was used to compare the responses i.e., “moderate or severe problems” to “no problem” for the sub-group analysis of each of the EQ5D domain. The analyses were adjusted for sociodemographic variables. A *p*-value of < 0.05 was considered statistically significant. All statistical analyses were performed using IBM SPSS for Windows Version 24 (IBM Corporation, Armonk, New York, USA).

## Results

Of the 410 clinic patients who were approached and screened, 46 patients were not eligible and 64 patients declined to participate in the study. Two participants were subsequently excluded as they did not fulfil the inclusion criteria and one participant participated twice on different days where the second survey by the same participant was excluded. A total of 297 participants were included in the study giving a response rate of 72.4%. Most of the participants were aged between 55 to 64 years, with a mean age of 56.6 ± 5.8 years. A portion of 41.7% had three or more conditions i.e. multimorbidity. The participants were mainly males (52.2%), Chinese (81.5%), married (78.1%), and employed (70.0%) (Table 1). The mean number of chronic conditions was 2.28 ± 1.2 and the most common conditions were lipid disorder (61.3%), hypertension (57.2%) and diabetes mellitus (34.3%).

**Table 1:** Demographic Characteristics of the participants (*N* = 297)

	N	(%)
**Age (years)**		
< 50	38	12.8
50–54	54	18.2
55–59	98	33.0
60–64	107	36.0
Mean (± SD)	56.6	(± 5.8)
Median (IQR)	58.0	(53.0–61.0)
**Sex**		
Male	155	52.2
Female	142	47.8
**Ethnicity**		
Chinese	242	81.5
Non-Chinese	55	18.5
**Main Spoken Language**		
English	92	31.0
Mandarin	148	49.8
Malay/Tamil/Others	57	19.2
**Marital Status**		
Married	232	78.1
Others^a^	65	21.9
**Education**		
Primary/No formal	89	30.0
Secondary	131	44.1
Post-Secondary	77	25.9
**Employed Or Not Employed**		
Employed	208	70.0
Unemployed/Retired	89	30.0
**Monthly Household Income**		
< $2000	60	20.2
$2000–$3999	78	26.3
$4000–$5999	54	18.2
≥ $6000	46	15.5
Not disclosed	59	19.9
**Dwelling**		
Institution/HDB 1–3 room	73	24.6
HDB 4 room	124	41.8
Other dwellings^b^	100	33.7
**Home Ownership**		
Owner-occupied	266	89.6
Not owner-occupied	31	10.4
**Living Arrangement**		
Living alone	20	6.7
Living with others	277	93.3

^a^Other marital status: single, widowed, divorced, and separated

^b^ Other dwellings: HDB 5-room or executive apartment, Housing and Urban Development Company apartment, HDB executive condominium, private condominium, and landed properties

*HDB* Housing and Development Board

All the participants completed the EQ5D questionnaire and the mean EQ5D UI and VAS were 0.843 ± 0.223 and 66.9 ± 16.4 respectively. There were 23 health states represented out of 243 possible ones from the study population. The most common (49.2%) EQ5D UI was 1.000, which corresponded to the best achievable health state of  "11111", representing “no problem” with all five domains. The lowest EQ5D UI was − 0.357 which corresponded to a health state of  "23222" i.e., “severe problems” with self-care and “moderate problems” with mobility, usual activities, anxiety/depression, and pain/discomfort. The EQ5D domain with the most reported problem was pain/discomfort (41.0%), followed by anxiety/depression (22.9%), mobility (13.5%), activities of daily living (9.8%), and self-care respectively (0.34%). Compared to participants with one or two conditions, those with three or more conditions, i.e. with multimorbidity, had higher odds (OR = 2.04, *p* = 0.01) of reporting problems with pain/discomfort (Table 2).

**Table 2:** Adjusted ORs of participants reporting “Moderate or Severe problems” across each of the 5 EQ5D domains

Mobility		Self-care		Activities of daily living		Pain / discomfort		Mood	
Adjusted OR (C.I)	*p*-value	Adjusted OR (C.I)	*p*-value	Adjusted OR (C.I)	*p*-value	Adjusted OR (C.I)	*p*-value	Adjusted OR (C.I)	*p*-value
1.41 (0.69–2.89)	0.35	-^c^	-^c^	1.37 (0.57–3.29)	0.48	2.04 (1.22–3.40)	0.01*	1.06 (0.58–1.91)	0.86

Adjusted for age, gender, ethnicity, language, marital status, education, employment, household income, dwelling, home ownership and living arrangement

^c^ Unadjusted OR for the domain of self-care domain is 13,133,941.80, as all participants without multimorbidity reported “No problems” with self-care, while only 1 participant with multimorbidity reported “Moderate problems” with self-care

*p-value < 0.05

Participants with three or more conditions, i.e. with multimorbidity, had lower EQ5D UI and VAS scores compared to those with one or two conditions. However, after adjusting for sociodemographic factors, only EQ5D UI was significantly associated with the number of chronic conditions (β-coefficient = − 0.064, *p* = 0.04) (Tables 3 and 4). In the same regression model, age and living arrangement were also associated with EQ5D UI. Participants aged less than 50 years old and those living with others had lower EQ5D UI compared to those aged 60 to 64 years old (β-coefficient = − 0.137, *p* = 0.01), and those living alone (β-coefficient = − 0.151, *p* = 0.02), respectively (Table 3).

**Table 3:** Unadjusted and adjusted means, and β coefficients of EQ5D UI across various predictors

Predictor Variables	Un adjusted mean	(SD)	Adjusted mean	(SD)	β	95% C.I	***p***-value
**No. of chronic condition(s)**							
1 or 2	0.871	(0.198)	0.837	(0.034)	REFERENCE	–	–
≥ 3	0.804	(0.251)	0.785	(0.032)	−0.064	(−0.125, − 0.003)	0.04*
**Age (years)**							
< 50	0.754	(0.304)	0.737	(0.042)	−0.137	(− 0.246, − 0.029)	0.01*
50–54	0.846	(0.217)	0.837	(0.042)	−0.01	(− 0.095, 0.075)	0.82
55–59	0.845	(0.222)	0.828	(0.036)	−0.021	(− 0.091, 0.050)	0.57
60–64	0.871	(0.187)	0.845	(0.036)	REFERENCE	–	–
**Sex**							
Male	0.843	(0.224)	0.801	(0.035)	REFERENCE	–	–
Female	0.843	(0.224)	0.82	(0.032)	0.023	(−0.040, 0.087)	0.48
**Ethnicity**							
Chinese	0.854	(0.212)	0.845	(0.034)	REFERENCE	–	–
Non-Chinese	0.795	(0.265)	0.777	(0.037)	−0.084	(−0.174, 0.005)	0.06
**Main spoken language**							
English	0.86	(0.249)	0.837	(0.036)	REFERENCE	–	–
Mandarin	0.835	(0.214)	0.778	(0.034)	−0.073	(−0.148, 0.001)	0.05
Malay/Tamil/ Others	0.838	(0.206)	0.817	(0.040)	−0.025	(− 0.116, 0.066)	0.59
**Marital status**							
Married	0.856	(0.206)	0.845	(0.038)	REFERENCE	–	–
Others^a^	0.797	(0.275)	0.777	(0.034)	−0.084	(− 0.171, 0.003)	0.06
**Education**							
Primary/ No formal	0.835	(0.218)	0.807	(0.038)	REFERENCE	–	–
Secondary	0.843	(0.239)	0.814	(0.036)	0.009	(−0.066, 0.083)	0.82
Post-secondary	0.851	(0.205)	0.811	(0.035)	0.005	(−0.087, 0.097)	0.91
**Employment**							
Employed	0.847	(0.217)	0.83	(0.032)	REFERENCE	–	–
Unemployed/ Retired	0.832	(0.239)	0.792	(0.036)	−0.047	(−0.116, 0.022)	0.18
**Monthly Household Income**							
< $2000	0.828	(0.284)	0.807	(0.037)	REFERENCE	–	–
$2000–$3999	0.861	(0.193)	0.831	(0.038)	0.03	(−0.059, 0.119)	0.51
$4000–$5999	0.808	(0.242)	0.791	(0.041)	−0.02	(− 0.120, 0.079)	0.69
≥ $6000	0.866	(0.186)	0.812	(0.045)	0.006	(−0.100, 0.113)	0.91
Not disclosed	0.848	(0.202)	0.811	(0.038)	0.005	(−0.090, 0.100)	0.92
**Dwelling**							
Institution/ HDB 1–3 room	0.841	(0.247)	0.824	(0.035)	REFERENCE	–	–
HDB 4 room	0.822	(0.222)	0.791	(0.036)	−0.041	(−0.122, 0.040)	0.32
Other dwellings^b^	0.87	(0.207)	0.817	(0.038)	−0.009	(− 0.096, 0.079)	0.85
**Home Ownership**							
Owner-occupied	0.855	(0.211)	0.842	(0.029)	REFERENCE	–	–
Not owner-occupied	0.743	(0.296)	0.78	(0.045)	−0.077	(−0.192, 0.037)	0.19
**Living arrangement**							
Living alone	0.919	(0.129)	0.874	(0.054)	REFERENCE	–	–
Living with others	0.837	(0.228)	0.751	(0.024)	−0.151	(−0.274, − 0.028)	0.02*

^a^Other marital status: not married, single, widowed, divorced, and separated

^b^ Other dwellings: HDB 5-room, HDB executive, Housing and Urban Development Company apartment, HDB executive condominium, private condominium, and landed properties

*HDB* Housing and Development Board

*p-value < 0.05

**Table 4:** Unadjusted and adjusted means, and β coefficients of EQ5D VAS across various predictors

Predictor Variables	Un adjusted mean	(SD)	Adjusted mean	(SD)	β	95% C.I	***p***-value
**No. of chronic condition(s)**							
1 or 2	68.5	(16.0)	69.6	(2.5)	REFERENCE	–	–
≥ 3	64.9	(16.8)	66.5	(2.5)	−0.045	(0.102, 0.12)	0.12
**Age (years)**							
< 50	62.8	(19.1)	63.2	(3.2)	−0.100	(− 0.199. -0.001)	0.05
50–54	68.8	(16.2)	71.4	(3.3)	0.021	(−0.058, 0.101)	0.60
55–59	66.5	(16.8)	68.0	(2.7)	−0.027	(−0.095, 0.041)	0.43
60–64	68.0	(15.0)	69.9	(2.7)	REFERENCE	–	–
**Sex**							
Male	66.8	(16.1)	67.8	(2.6)	REFERENCE	–	–
Female	67.1	(16.7)	68.3	(2.4)	0.006	(0.053, 0.066)	0.84
**Ethnicity**							
Chinese	66.8	(16.2)	67.4	(2.5)	REFERENCE	–	–
Non-Chinese	67.9	(17.5)	68.7	(2.9)	0.019	(0.064, 0.101)	0.66
**Main Spoken Language**							
English	67.4	(16.3)	69.4	(2.8)	REFERENCE	–	–
Mandarin	66.4	(16.4)	66.8	(2.7)	−0.038	(−0.110, 0.034)	0.31
Malay/Tamil/ Others	67.8	(16.9)	68.0	(3.1)	−0.021	(−0.106, 0.065)	0.64
**Marital Status**							
Married	67.3	(16.0)	68.7	(2.8)	REFERENCE	–	–
Others^a^	65.8	(18.0)	67.4	(2.6)	−0.018	(−0.097, 0.061)	0.65
**Education**							
Primary/ No formal	67.8	(16.4)	70.1	(3.0)	REFERENCE	–	–
Secondary	67.3	(15.7)	68.6	(2.8)	−0.022	(−0.091,0.048)	0.54
Post-secondary	65.5	(17.7)	65.5	(2.6)	−0.068	(− 0.156, 0.020)	0.13
**Employment**							
Employed	67.1	(16.5)	68.6	(2.4)	REFERENCE	–	–
Unemployed/ Retired	66.7	(16.3)	67.5	(2.7)	−0.016	(−0.081, 0.049)	0.62
**Monthly Household Income**							
< $2000	64.7	(18.0)	64.8	(2.8)	REFERENCE	–	–
$2000–$3999	68.8	(14.6)	70.4	(2.9)	0.083	(−0.002, 0.168)	0.06
$4000–$5999	66.0	(18.0)	68.2	(3.2)	0.051	(−0.042, 0.144)	0.28
≥ $6000	68.6	(14.9)	70.7	(3.6)	0.087	(−0.015, 0.189)	0.09
Not disclosed	66.5	(16.7)	66.4	(2.9)	0.025	(−0.066, 0.116)	0.59
**Dwelling**							
Institution/ HDB 1–3 room	68.9	(19.0)	70.0	(2.7)	REFERENCE	–	–
HDB 4 room	65.1	(16.1)	65.9	(2.7)	−0.061	(−0.135, 0.013)	0.11
Other dwellings^b^	67.9	(14.6)	68.3	(2.9)	−0.026	(− 0.106, 0.054)	0.53
**Home Ownership**							
Owner-occupied	67.2	(15.8)	68.9	(2.3)	REFERENCE	–	–
Not owner-occupied	64.7	(21.2)	67.2	(3.4)	−0.026	(−0.127, 0.076)	0.62
**Living Arrangement**							
Living alone	70.3	(21.7)	71.0	(4.1)	REFERENCE	–	–
Living with others	66.7	(16.0)	65.2	(1.9)	−0.086	(−0.204, 0.033)	0.16

^a^Other marital status: not married, single, widowed, divorced, and separated

^b^ Other dwellings: HDB 5-room, HDB executive, Housing and Urban Development Company apartment, HDB executive condominium, private condominium, and landed properties

*HDB* Housing and Development Board

*p-value < 0.05

## Discussion

We conducted a cross-sectional study investigating the association between multimorbidity and HrQoL in middle-aged patients at a primary care setting in Singapore. In the study, participants with multimorbidity had significantly lower domain-specific HrQoL scores (EQ5D UI) but not global HrQoL scores (VAS). Specifically, participants reported more problems with the domain of pain/discomfort.

Middle-aged patients with multimorbidity had lower EQ5D UI, and is in keeping with the findings from other studies [9, 11, 36]. Multimorbidity increases the disease burden and affects one’s HrQoL. Additionally, patients with multimorbidity are more likely to experience higher treatment burden which includes polypharmacy, adjustment to major lifestyle changes, constant monitoring of one’s own health status, and navigation of a complex healthcare system [26, 37, 38]. Middle-aged adults also often have multiple financial and care-giving responsibilities [19, 21] which may be overwhelming for them to balance these responsibilities with their own healthcare needs.

Although patients with multimorbidity had lower EQ5D VAS in our study, the association was not statistically significant. This is in contrast to other studies in the primary care [39] and general populations [40] that reported an inverse relationship between multimorbidity and VAS scores. The EQ5D UI is based on the participants’ selection of one out of three responses to each of the five EQ5D domains that is weighted by general public preferences. In contrast, the VAS is derived from the participants’ self-indication of their general health for that day. Compared to the choice-based UI, the VAS measures a broader construct of the individual’s health which is not confined to the five specific domains and is more reflective of the individual’s perception of his or her own general health state [41]. This study suggested that while multimorbidity was associated with poorer HrQoL compared to those without multimorbidity, as measured by a composite of pain, physical functioning, and mental wellbeing in the middle-aged participants, it was not associated with the general health state. When considering their general health state, the participants could have perceived their chronic conditions as mild, with little impact on their lives. While illness perception has been associated with HrQoL in single diseases, [42, 43] its influence on HrQoL in patients with multimorbidity is not well studied. Further studies are required to understand the association of patients’ illness perceptions with multimorbidity and their HrQoL.

Another interesting finding in our study was the significant association between multimorbidity and the EQ5D domain of pain/discomfort. The domain of pain/discomfort has the highest percentage of reported problems in our study and this is similar to other studies [12, 36, 39, 44] Chronic pain is a common, complex, and challenging condition and the extent to which multimorbidity is associated with chronic pain in the middle-aged population is unknown. A cross-sectional analysis of the elderly MultiCare Cohort Study sample found that chronic pain, as measured by the Graded Chronic Pain Scale [45] was largely associated with chronic lower back problems [17] In our study, we were not able to distinguish if participants who reported problems to pain/discomfort had chronic or acute pain, neither were we able to determine the cause(s) of the participants’ pain/discomfort. One may suffer from pain caused by the side-effects of medications, or from the discomfort caused by the disease(s). Moreover, chronic pain is strongly influenced by demographic and psychosocial factors [46] Future studies may be undertaken to evaluate the factors contributing to chronic pain and HrQoL in middle-aged patients with multimorbidity. This can contribute to subsequent interventions to improve HrQoL in this population.

One significant observation is that our sample is slightly older compared to other middle-aged primary care populations, [3, 15, 24] with most of our patients aged between 55 and 64 years old. This is reflective of the middle-age distribution at our centre as well as the fast-ageing Singaporean population [22] Within our study population participants below 50 years of age had poorer HrQoL compared to those aged 60 years and above, a finding similar to that by Peters et al. [47] Possible explanations include the burden of additional responsibilities such as work or caring for children and elderly parents, and the higher likelihood of younger people reporting mental health problems which may have affected the EQ5D UI [47]. However, the adaptability of patients to the onset of new conditions and different chronic disease trajectories may change with time [47]. We also found that participants living with others had lower HrQoL compared to those living alone. Middle-aged patients with multimorbidity may face additional stress from caring for dependents, who would most likely be staying with them. Prazeres et al. [48] also showed that living arrangement may affect both the physical and mental components of HrQoL in patients with multimorbidity.

Finally, it is important to note that we used a higher cut-off of three or more conditions to define multimorbidity in this study. Patients with two chronic conditions that are considered as multimorbid in other studies are considered as non-multimorbid in our study. Although there is currently no standardized definition of multimorbidity, most authors use a cut-off of two or more conditions [28]. There are exceptions with some studies [11, 18] using a cut-off of three or more, especially when the prevalence of multimorbidity in the study population is high. Harrison et al. [4] found that by using a cut-off of two or more conditions, one in two primary care patients would be diagnosed with multimorbidity, whereas using a higher cut-off of three or more would decrease the estimate to one in four. This was also reflected in an unpublished study in our local primary care setting [29]. Using a lower cut-off may identify such a high proportion of patients as having multimorbidity that the measure lacks specificity to be useful, [2, 4] hence we decided on a higher cut-off as this will enable us to identify patients with higher needs.

### Strengths and limitations

This study has a few limitations. Firstly, the cross-sectional nature does not allow establishment of causal relationships. Secondly, as the study was done at a single polyclinic, there was slight over-representation of Chinese and under-representation of Malays and Indians compared to the national population. Thirdly, the data collected were self-reported and there may be under or over-reporting of chronic medical conditions [49, 50] Fourthly, we did not include patients with no chronic conditions in our study. The focus of our study was on how having chronic conditions can affect HrQoL and such patients often present at our clinic with acute self-limiting conditions [25] which may transiently affect their HrQoL without any meaningful long term impact.

The strengths of our study include the administration of the study questionnaires in multiple languages to maximize sample representativeness, and the selection of a validated HrQoL measure with local HrQoL weights. In addition, our study used a systematic randomized sampling method to select potential participants as an attempt to better represent the primary care population.

## Conclusion

This study showed that multimorbidity, as measured by a count of chronic conditions, was not associated with self-perceived global HrQoL but was negatively associated with domain specific HrQoL, specifically for the domain of pain/discomfort. Further studies exploring chronic pain in the middle-aged primary care population with multimorbidity can help physicians better manage pain and improve the HrQoL in this population.

## Data Availability

The datasets generated and/or analysed during the current study are not publicly available but are available from the corresponding author on reasonable request.

## Abbreviations

EQ5D-3L: EuroQol 5 dimensions -3 levels
HDB: Housing Development Board
HrQoL: Health-related quality of life
UI: Utility Index
VAS: Visual Analogue Scale
